# Pemphigus Vulgaris Mimicking Behçet’s Syndrome With Oral and Vulvar Ulcers: A Case Report

**DOI:** 10.7759/cureus.101772

**Published:** 2026-01-18

**Authors:** José N Magalhães, Diogo Carvalho Sá

**Affiliations:** 1 Medicine Department, Unidade Local de Saúde de Santo António, Porto, PRT; 2 Pathology Department, Unidade Local de Saúde de Santo António, Porto, PRT

**Keywords:** behçet's syndrome, mucosal pemphigus, oral erosions, pemphigus vulgaris, vulvar erosions

## Abstract

Recurrent oral and genital ulceration commonly raises suspicion for Behçet’s syndrome, but the oral-genital pattern is not specific and should prompt a structured differential diagnosis, particularly in mucosa-predominant presentations without systemic features. We report a 62-year-old woman referred for a four-month history of synchronous, painful oral and vulvar mucosal lesions associated with odynophagia/dysphagia and 9% unintentional weight loss. She had no ocular symptoms, no cutaneous lesions, and routine laboratory testing was globally unremarkable. Behçet’s syndrome was initially considered, and empiric therapy with colchicine followed by systemic corticosteroids was instituted without clinical improvement. An oral mucosal biopsy was therefore performed, and histopathology with direct immunofluorescence established the diagnosis of pemphigus vulgaris. This case highlights pemphigus vulgaris as a clinically plausible Behçet mimic when oral and genital mucosal disease coexist, and underscores the value of timely biopsy with immunofluorescence in refractory oral-genital erosive disease.

## Introduction

Behçet’s syndrome is a multisystem inflammatory disorder in which recurrent oral aphthosis and genital ulceration are common and may lead to early diagnostic anchoring, despite the absence of a single pathognomonic test and the need for a structured differential diagnosis in mucosa-predominant presentations [1,2]. Accordingly, the differential diagnosis of recurrent oral-genital erosive/ulcerative disease is broad and includes inflammatory mucosal disorders and autoimmune blistering disease, notably pemphigus vulgaris and mucous membrane pemphigoid [3]. Because diagnosis is clinical, classification criteria such as the International Criteria for Behçet’s Disease (ICBD) can support structured reasoning by weighting key mucocutaneous, ocular, vascular, and neurologic domains [2]. Worldwide prevalence varies markedly, clustering along the “Silk Road” and reaching the highest reported rates in Turkey, with typical onset in early adulthood [4]. Pemphigus vulgaris is an autoimmune blistering disease typically presenting in mid-life, with varying incidence across populations and higher rates reported in some ethnic groups, including individuals of Ashkenazi Jewish and Mediterranean ancestry [5]. Mucosal involvement is frequent and may precede or occasionally occur without cutaneous lesions; genital involvement, historically considered uncommon, is increasingly recognized and can present with non-specific vulvar erosions, thereby mimicking other inflammatory disorders when oral and genital lesions coexist [6-8]. In patients with oral-genital erosive disease that fails to improve under an initial Behçet-directed approach, early biopsy becomes the diagnostic pivot that centers the case on clinicopathologic correlation and redirects management toward disease-specific care [6,9,10]. Here, we report a woman referred for recurrent oral and vulvar lesions initially suspected as Behçet’s syndrome, in whom biopsy confirmed pemphigus vulgaris.

## Case presentation

A 62-year-old White woman, with no relevant past medical history and no regular medication, was referred to the Internal Medicine outpatient clinic for a four-month history of synchronous, recurrent painful oral and vulvar mucosal lesions, progressively limiting oral intake. She reported odynophagia/dysphagia driven by oral pain and an unintentional 9% weight loss over the same period. There was no prior history of recurrent oral aphthae before this four-month episode. Family history was unremarkable. She reported no high-risk sexual exposure and no recent new sexual partners. She denied ocular symptoms, cutaneous blisters or lesions, and other systemic manifestations.

**Figure 1:**
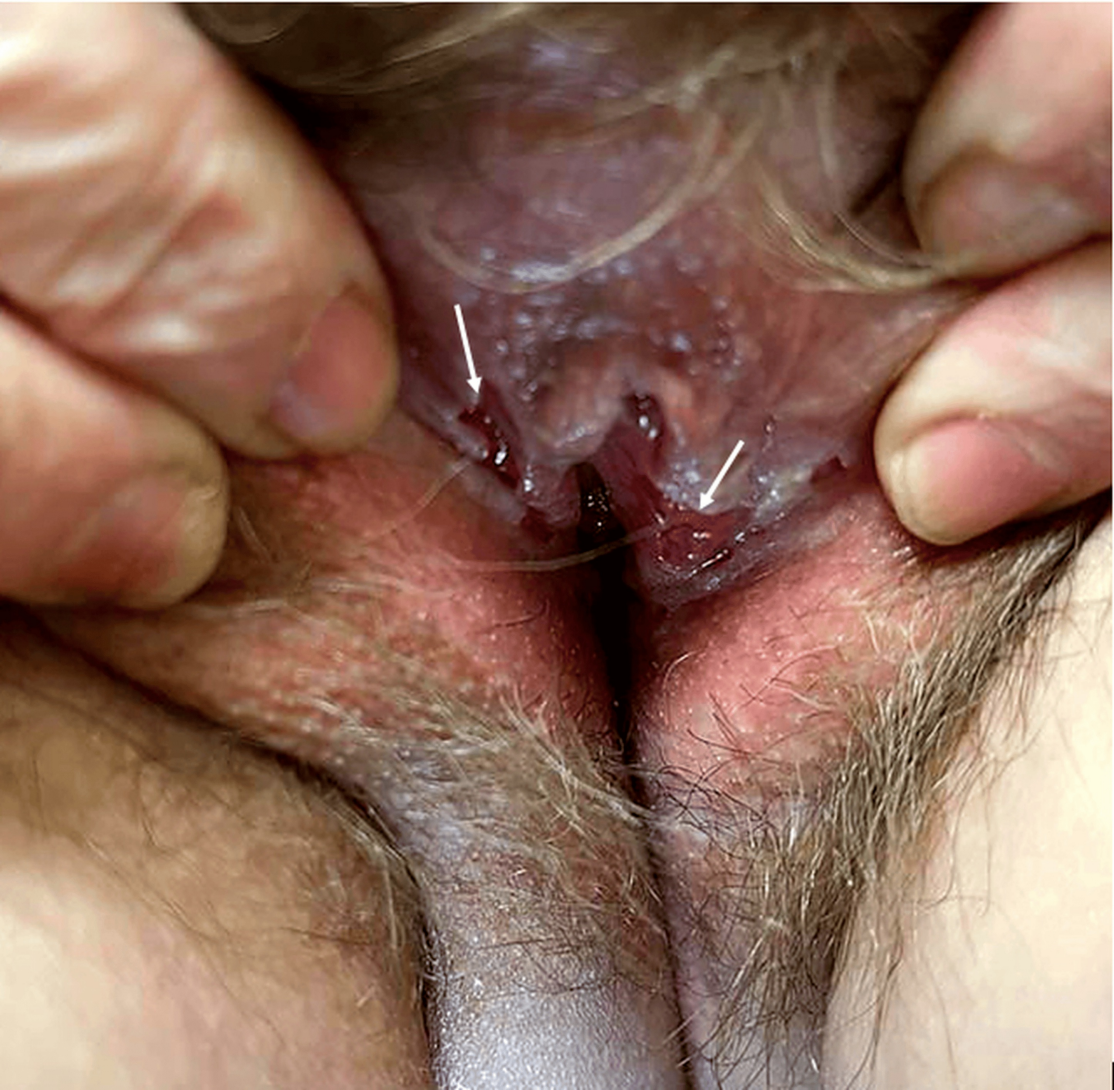
Vulvar involvement showing erythematous erosions with focal exudation, predominantly affecting the inner aspect of the labia and vestibule. White arrows indicate the most representative lesions.

Oral examination disclosed multiple shallow erosions on the dorsal tongue, some with a whitish surface slough, and surrounding mucosal erythema. Additional erosive/bullous-appearing lesions were noted on the soft palate and tonsillar pillars. Genital examination showed vulvar mucosal erosions with exudative areas, at the inner labial/vestibular mucosa, without scarring or architectural distortion (Figure 1).

Baseline investigations, including complete blood count, renal and liver function tests, and C-reactive protein, were within normal limits, apart from a mildly elevated erythrocyte sedimentation rate. Pathergy testing was not performed. An autoimmune work-up performed during the initial assessment was negative (Table 1).

**Table 1:** Initial laboratory assessment. ANA (IIF): antinuclear antibodies by indirect immunofluorescence

Parameter	Result	Units	Normal range
White blood cells	8.88	x10^3/µL	4.00-11.00
Neutrophils (%)	42.9	%	40.0-75.0
Lymphocytes (%)	47.2	%	20.0-45.0
Monocytes (%)	7.0	%	2.0-10.0
Eosinophils (%)	2.1	%	1.0-6.0
Basophils (%)	0.6	%	0.0-1.0
Erythrocytes	4.28	x10^6/µL	3.8-4.8
Hemoglobin	13.0	g/dL	12.0-15.0
Platelets	358	x10^3/µL	150-400
Sedimentation rate (first hour)	43	mm	0-20
Urea	50	mg/dL	10-50
Creatinine	0.76	mg/dL	0.5-0.9
Albumin	4.25	g/dL	3.4-4.8
Total proteins	6.63	g/dL	6.0-7.3
C-reactive protein	3.01	mg/L	0.0-5.0
IgA	163	mg/dL	114-457
IgG	964	mg/dL	793-1590
IgM	95.7	mg/dL	29.0-226.0
C3	133.0	mg/dL	81.0-167.9
C4	28.8	mg/dL	11.0-42.0
ANA (IIF) titer	1/80	-	<1/160

**Figure 2:**
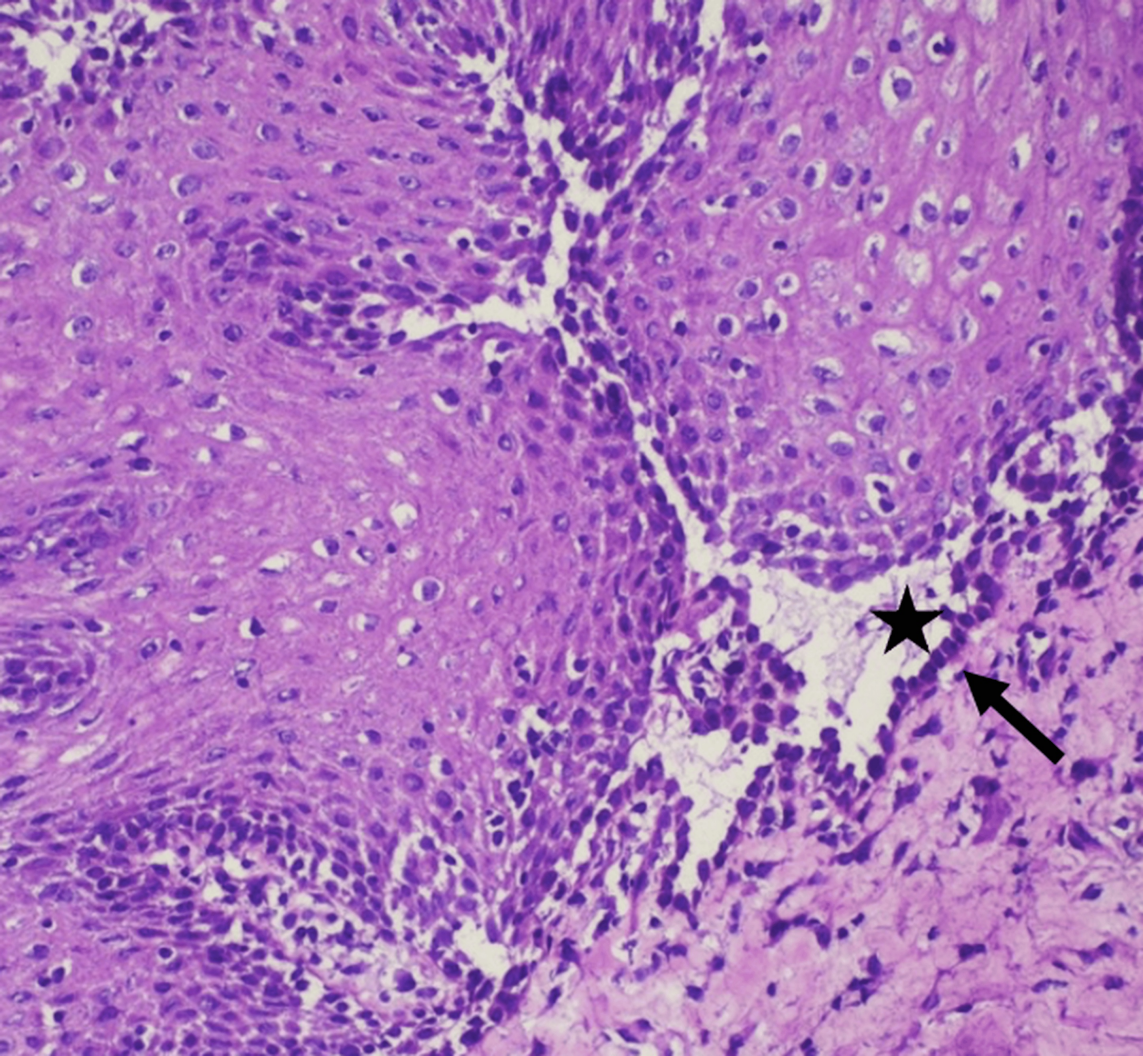
Histopathology (H&E, 200×) showing suprabasal clefting (star) with acantholysis; basal keratinocytes remain attached to the basement membrane, producing a “tombstone” appearance (arrow).

**Figure 3:**
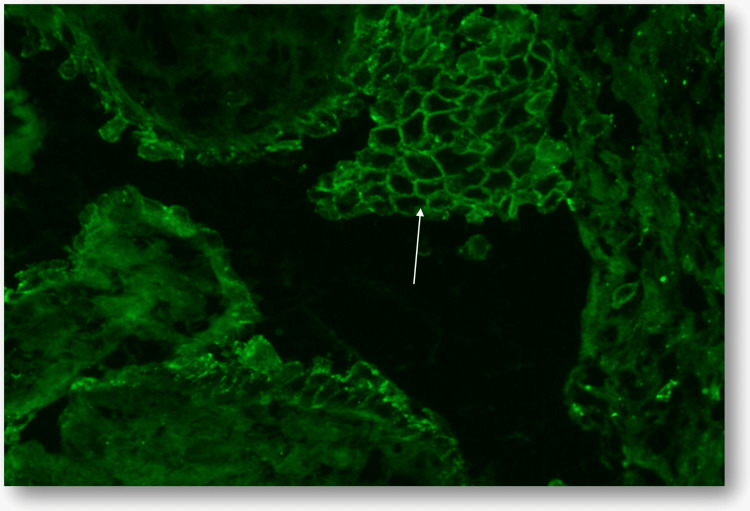
Direct immunofluorescence for C3 demonstrating intercellular granular deposition in a reticular “fishnet” pattern (arrow).

Given the oral-genital pattern, Behçet’s syndrome was considered, and colchicine was initiated, followed by systemic corticosteroids due to persistent symptoms, without clinical improvement. An oral mucosal biopsy was therefore performed. Histopathology showed suprabasal clefting with acantholysis, and direct immunofluorescence demonstrated a reticular intercellular deposition pattern, establishing the diagnosis of pemphigus vulgaris (Figures 2, 3).

After histologic confirmation, a directed autoimmune blistering disease work-up was requested and demonstrated anti-desmoglein 3 antibody positivity, providing serologic support for the diagnosis (Table 2).

**Table 2:** Directed autoimmune blistering disease work-up after histologic diagnosis. IIF: indirect immunofluorescence; ELISA: enzyme-linked immunosorbent assay

Parameter	Result	Units	Normal range
Basement membrane antibodies (IIF)	Negative	-	<1/10
Anti-BP180 (ELISA)	Negative	-	Negative
Anti-BP230 (ELISA)	Negative	-	Negative
Anti-envoplakin (ELISA)	Negative	-	Negative
Anti-collagen VII (ELISA)	Negative	-	Negative
Anti-desmoglein 1 (ELISA)	Negative	-	Negative
Anti-desmoglein 3 (ELISA)	Positive	-	Negative
Anti-desmoglein 3 (ELISA)	153.5	U/mL	<20

## Discussion

The co-occurrence of recurrent oral and genital lesions readily activates a Behçet-centered diagnostic frame. In this setting, the 1990 International Study Group (ISG) criteria define Behçet’s disease as recurrent oral ulceration plus at least two of the following: recurrent genital ulceration, eye lesions, skin lesions, or a positive pathergy test [11]. Nevertheless, these criteria do not replace clinical judgement, as Behçet’s syndrome remains a clinical diagnosis without a single pathognomonic diagnostic test, and classification frameworks such as the ICBD support structured reasoning by weighting clinical domains and using a score threshold for classification [2,12]. Therefore, in mucosa-predominant presentations, diagnostic plausibility depends on the broader constellation of findings and on the clinical trajectory under initial therapy rather than on the oral-genital pattern alone [1,2]. In the present case, the synchronous oral-genital pattern supported Behçet’s as an initial working hypothesis and justified a time-limited directed therapeutic trial while the differential remained open. The absence of ocular involvement, cutaneous lesions, or other systemic features, together with a lack of response to a Behçet-directed approach, supported an early shift from syndromic reasoning to tissue diagnosis.

Pemphigus vulgaris provides a coherent alternative explanation because mucosal involvement is frequent, oral disease may precede cutaneous manifestations, and mucosal fragility typically translates clinically into erosions rather than intact bullae [6,7]. Genital involvement, previously considered uncommon, has been documented in dedicated cohorts and may present as non-specific vulvar erosions, increasing the overlap with Behçet-like phenotypes when oral and genital disease coexist. Skin lesions are absent [8].

In practical terms, biopsy complemented by direct immunofluorescence should be regarded as an early pivot in refractory oral-genital erosive disease, because it establishes diagnosis with high specificity and prevents prolonged exposure to non-targeted immunosuppression trajectories [9,10]. In this case, the combination of suprabasal acantholysis on routine histology and a reticular intercellular deposition pattern on immunofluorescence provided diagnostic closure and redirected management toward pemphigus-specific care.

## Conclusions

Concurrent oral and genital mucosal erosions can anchor diagnostic reasoning in Behçet’s syndrome, particularly when the presentation is mucosa-predominant and systemic features are absent. This case illustrates that pemphigus vulgaris may present with a Behçet-like oral-genital pattern and should be considered when the clinical course is refractory to initial Behçet-directed treatment. In this setting, a timely biopsy with histopathology and direct immunofluorescence provides diagnostic closure and redirects management toward disease-specific therapy.
